# Tetralogy of fallot in addition to anomalous aortic origin of a coronary artery in a 1-year-old boy: a case report

**DOI:** 10.1186/s12893-021-01380-3

**Published:** 2021-10-30

**Authors:** Lihua Deng, Tiange Li, Yunfei Ling, Menglin Tang

**Affiliations:** 1 Department of Intensive Care Unit, West China Hospital, Sichuan University, No. 37 GuoXue Xiang, Chengdu, 610041 Sichuan People’s Republic of China; 2 Department of Cardiovascular Surgery, West China Hospital, Sichuan University, No. 37 GuoXue Xiang, Chengdu, 610041 Sichuan People’s Republic of China

**Keywords:** Anomalous aortic origin of a coronary artery (AAOCA), Tetralogy of Fallot (TOF), Infant, Case report

## Abstract

**Background:**

Anomalous aortic origin of a coronary artery (AAOCA) is a rare congenital heart disease, characterized by the coronary artery inappropriately originates from the aorta. It is usually classified according to the sinus where the coronary artery arises from, while anomalous origin of the right coronary being the most common type.

**Case presentation:**

In this case report, we described a rare case of Tetralogy of Fallot (TOF) in a 1-year-old boy, who also had the anomalous right coronary artery that originated from the left coronary sinus without an intramural segment. Besides TOF repair, lateral pulmonary translocation was undertaken in order to avoid risks of myocardial ischemia.

**Conclusion:**

We successfully completed a one-stage operation consisting of TOF repair and pulmonary artery translocation in a 1-year-old boy. We advocated early operation of pulmonic translocation for AAOCA patients without an intramural segment instead of unroofing procedure.

## Background

Anomalous aortic origin of a coronary artery (AAOCA) is a rare congenital heart condition, in which the origin of a coronary artery that arises from the aorta is abnormal, and the coronary artery usually has an intramural segment [1]. The prevalence of AAOCA is uncertain, probably ranges from 0.21 to 5.79% in the general population [2–4]. While in those TOF patients, it seems not so rare, accounts for about 10% [5–7]. Although rare, AAOCA is recognized as the second leading cause of sudden cardiac death (SAD) in children and adolescents. AAOCA is usually classified according to the location of the anomalous coronary ostium: right coronary artery (RCA) originates from the left coronary sinus, left coronary artery (LCA) originates from the right coronary sinus, or, one or both coronary arteries originate from the non-coronary sinus.

All types of AAOCA are associated with increased risks of myocardial ischemia and SAD, especially in young athletes [8]. Studies have reported that the anomalous origin of RCA is more common than the anomalous origin of LCA [9], while the anomalous origin of LCA carries a higher risk of SAD [10]. The most common surgery performed for patients with an AAOCA and an intramural segment is the unroofing procedure of the intramural segment, which may reduce the risk of SAD during physical activities [11]. On the other hand, lateral pulmonary translocation can be performed for those without an intramural segment [12]. To our knowledge, this is the first case report in the literature that described a rare presentation of a 1-year-old boy with both TOF and AAOCA who successfully underwent a combined surgical procedure of TOF with pulmonary artery translocation.

## Case presentation

**Fig. 1:**
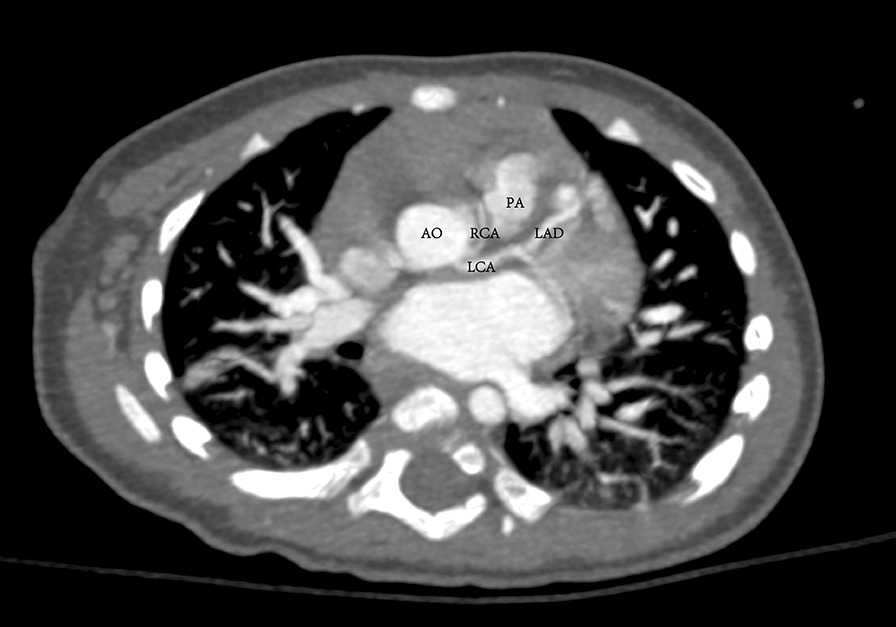
Computed tomography (CT) revealed both anomalous RCA and the normal LCA arose from the left coronary sinus, which coursed between the aorta and the pulmonary artery without an intramural segment

**Fig. 2:**
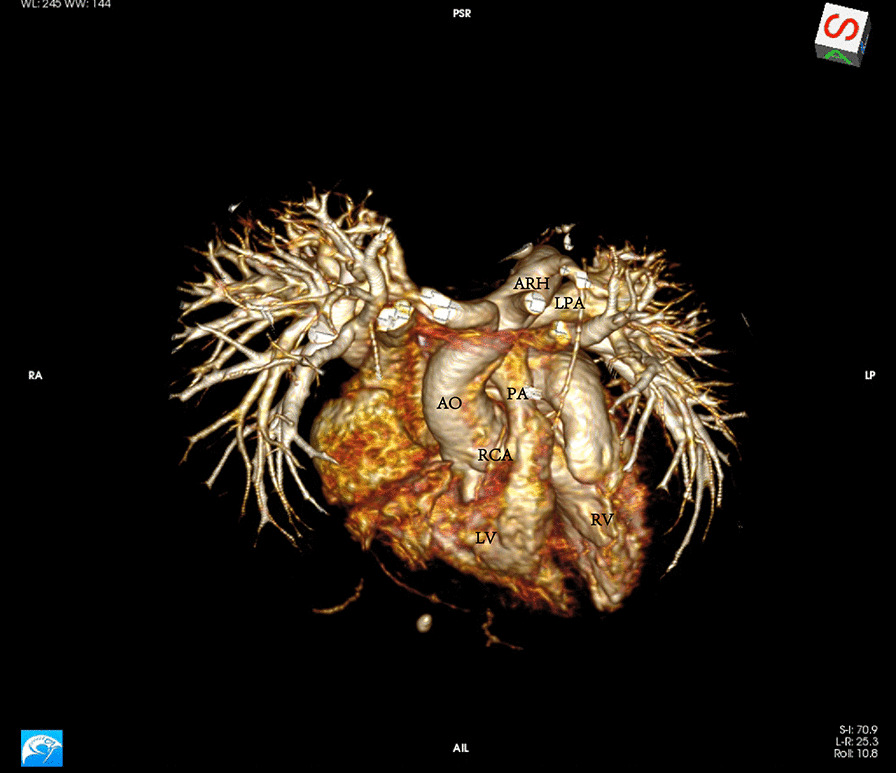
Narrow pulmonary trunk with normal left and right pulmonary artery

A 1-year-old boy presented to our center from a regional hospital for further assessment and management of TOF. The child was found to have structural abnormalities of the heart in his fetal stage. He presented with mild cyanosis, slight shortness of breath after activity, and poor feeding. Physical examination revealed a normal growth level at the height of 78 cm and the weight of 8.5 kg. On further investigation, the transoesophageal echocardiography (TEE) demonstrated the common features of TOF consisting of a 14-mm ventricular septal defect (VSD), pulmonary valve stenosis with a 5-mm valve ring, supravalvular pulmonary stenosis with a 4-mm local inner diameter, subvalvular pulmonary stenosis with a 5-mm infundibular pulmonary artery, 40% overriding of the aorta above VSD, and 6-mm-thickness right ventricular anterior wall. The Color Doppler assessment demonstrated a shunt flow mainly from the left ventricle to the right at the level of VSD, pulmonary valve stenosis and supravalvular pulmonary stenosis with a pressure gradient of 106 mmHg, infundibular pulmonary artery stenosis with a pressure gradient of 78 mmHg, and mild mitral and tricuspid regurgitation. Further assessment with computed tomography (CT) not only confirmed the diagnosis of TOF but also revealed both anomalous RCA and the normal LCA arose from the left coronary sinus, which coursed between the aorta and the pulmonary artery without an intramural segment (Fig. 1). Additionally, the CT also demonstrated stenosis of the pulmonary trunk and the normal left and right pulmonary artery (Fig. 2).

Given the clinical features and radiological findings, a one-stage operation was carried out, which consisted of TOF repair (including VSD closure, trans-annular enlargement of the right ventricular outflow tract, and infundibular muscle resection) and lateral pulmonary translocation that aimed to improve coronary flow. A median sternotomy was performed, and cardiopulmonary bypass was initiated through aortic and bi-caval cannulation. Antegrade cardioplegia was administrated to induce cardiac arrest. TOF repair was first performed, followed by pulmonary artery translocation by introducing an autologous blood vessel patch of the right pulmonary artery and inserting this patch in the lateral aspect of the main pulmonary artery. Postoperatively, the TEE showed no residual shunting, mitral or tricuspid regurgitation, with the CT revealed normal blood flow of RCA without inter-arterial compression. The boy was stable during the immediate postoperative period with no arrhythmia or cyanosis. He recovered uneventfully and was discharged from the hospital.

## Discussion and conclusion

Being a rare congenital cardiac disease, the reported prevalence of AAOCA in the general population varies widely, with the true incidence remains unknown [13]. As previously mentioned, the prevalence of AAOCA in TOF patients is higher than that in the general population. Compared to single AAOCA, combined with TOF may influence the selection of surgical procedures. However, recognized AAOCA in TOF patients is still a challenge, some studies reported that postmortem examinations indicate a higher incidence [14, 15]. Thus, reasonable screening and careful evaluation are advisable in TOF patients preoperatively. Selective coronary angiography was considered as a “golden standard” method to diagnose AAOCA, nevertheless, it is not a suitable method for screening. As a noninvasive method, echocardiographic screening was recommended to recognize AAOCA in TOF patients recently study [16].

The clinical manifestations of patients with AAOCA also vary, ranging from complete lack of symptoms to obvious myocardial ischemia such as chest pain and SAD, though the exact mechanisms and the crucial risk factors leading to SAD have not been well understood [17]. An increased cardiac output due to exercise or stress, leading to compression of the abnormal coronary artery between the aorta and pulmonary artery has been regarded previously as the main reason for myocardial ischemia and SAD [18]. Recently, studies have found that factors including intramural course, acute take-off angle, slit-like ostium and proximal narrowing with elliptic vessel shape may be accounted for the occurrence of myocardial ischemia and SAD [1, 19–22]. Nevertheless, AAOCA is associated with an increased risk of SAD and thus, corrective surgery is warranted to prevent adverse outcomes.

As the most accepted surgical procedure for AAOCA with intramural segments, unroofing could reduce the incidence of SAD. Alternatively, translocation and reimplantation can be performed for those without intramural segments [23], for this procedure could effectively relieve the compression of anomalous coronary artery. On the other hand, enlargement of the pulmonary artery is an essential component during TOF repair, which may result in the possibility of inter-arterial compression of the RCA. To achieve the optimal outcome for our patient, a one-stage operation consisted of TOF repair and pulmonary artery translocation was conducted to correct both the TOF and AAOCA, respectively. Another study has described the procedure named ostioplasty, which was less utilized but could be selected for AAOCA patients without acute take-off angle or commissural ostial location [24].

In summary, our case has demonstrated the feasibility and success of a one-stage operation consisting of TOF repair and pulmonary artery translocation for a patient presented with both the TOF and anomalous RCA that arose from the left coronary sinus without an intramural segment. Although rare, patients with such complex congenital heart disease should be considered for simultaneous repair of all the anomalies for the best possible long-term outcome.

## Data Availability

Data sharing is not applicable to this article as no datasets were generated or analyzed during the current study.

## Abbreviations

AAOCA: Anomalous aortic origin of a coronary artery
TOF: Tetralogy of Fallot
SAD: Sudden cardiac death
RCA: Right coronary artery
LCA: Left coronary artery
